# Mechanistic Role of Reactive Oxygen Species and Therapeutic Potential of Antioxidants in Denervation- or Fasting-Induced Skeletal Muscle Atrophy

**DOI:** 10.3389/fphys.2018.00215

**Published:** 2018-03-14

**Authors:** Jiaying Qiu, Qingqing Fang, Tongtong Xu, Changyue Wu, Lai Xu, Lingbin Wang, Xiaoming Yang, Shu Yu, Qi Zhang, Fei Ding, Hualin Sun

**Affiliations:** ^1^Laboratory of Neuroregeneration, Jiangsu Clinical Medicine Center of Tissue Engineering and Nerve Injury Repair, Co-Innovation Center of Neuroregeneration, Nantong University, Nantong, China; ^2^School of Medicine, Nantong University, Nantong, China

**Keywords:** skeletal muscle atrophy, reactive oxidative species, microarray, antioxidant therapy, N-acetyl-L-cysteine, pyrroloquinoline quinone

## Abstract

Skeletal muscle atrophy occurs under various conditions, such as disuse, denervation, fasting, aging, and various diseases. Although the underlying molecular mechanisms are still not fully understood, skeletal muscle atrophy is closely associated with reactive oxygen species (ROS) overproduction. In this study, we aimed to investigate the involvement of ROS in skeletal muscle atrophy from the perspective of gene regulation, and further examine therapeutic effects of antioxidants on skeletal muscle atrophy. Microarray data showed that the gene expression of many positive regulators for ROS production were up-regulated and the gene expression of many negative regulators for ROS production were down-regulated in mouse soleus muscle atrophied by denervation (sciatic nerve injury). The ROS level was significantly increased in denervated mouse soleus muscle or fasted C2C12 myotubes that had suffered from fasting (nutrient deprivation). These two muscle samples were then treated with N-acetyl-L-cysteine (NAC, a clinically used antioxidant) or pyrroloquinoline quinone (PQQ, a naturally occurring antioxidant), respectively. As compared to non-treatment, both NAC and PQQ treatment (1) reversed the increase in the ROS level in two muscle samples; (2) attenuated the reduction in the cross-sectional area (CSA) of denervated mouse muscle or in the diameter of fasted C2C12 myotube; (3) increased the myosin heavy chain (MHC) level and decreased the muscle atrophy F-box (MAFbx) and muscle-specific RING finger-1 (MuRF-1) levels in two muscle samples. Collectively, these results suggested that an increased ROS level was, at least partly, responsible for denervation- or fasting-induced skeletal muscle atrophy, and antioxidants might resist the atrophic effect via ROS-related mechanisms.

## Introduction

Skeletal muscle is a very important organ in the body and maintains many important functions, such as locomotion, metabolism, and respiration. However, diverse physio-pathological stimuli, including disuse, denervation, fasting, aging, and systematic diseases, can trigger skeletal muscle atrophy, which is defined as muscle mass loss and muscle function impairment resulting from an increase in muscle protein degradation and a decrease in protein synthesis (Bodine and Baehr, 2014; Huang and Zhu, 2016). Skeletal muscle atrophy has devastating effects on patients' quality of life and survival, and so understanding its molecular basis and developing countermeasures to block or attenuate the atrophic process has become an active area of intensive research (Cohen et al., 2015).

The ubiquitin-proteasome pathway (UPS) is one of the main intracellular proteolysis systems (Fanzani et al., 2012). The UPS is believed to be involved with the control of skeletal muscle atrophy, serving as one of the major proteolytic machineries in different muscle models (Bodine and Baehr, 2014; Polge et al., 2015; Wing, 2016). For example, muscle RING finger-1 (MuRF1) and muscle atrophy F-box (MAFbx)/atrogin-1 are two muscle-specific E3 ubiquitin ligases of the UPS (Bodine and Baehr, 2014; Winbanks et al., 2016). As early as 2001, two pioneering studies reported that MuRF1 and MAFbx genes were similarly altered in disparate muscle atrophy conditions (Bodine et al., 2001; Gomes et al., 2001). Among a number of later works, our observations showed that skeletal denervation- or drug-induced skeletal muscle atrophy led to significant up-regulation of MuRF1 and MAFbx (Sun et al., 2014a,b; He et al., 2016), while other researchers found that MuRF1 and MAFbx were up-regulated in skeletal muscle atrophy associated with cancer cachexia and cerebral ischemia (Gomes et al., 2012; Desgeorges et al., 2015; Winbanks et al., 2016).

Despite advances in elucidating molecular aspects of skeletal muscle atrophy, the intracellular signaling pathways involved are still being vigorously explored. Reactive oxygen species (ROS) play important roles in normal regulation of many physiological processes. However, the abnormal production of ROS in response to environmental stressors may lead to widespread cell damage (Tong et al., 2015; Zuo et al., 2015a,b). In this respect, the mechanistic links between ROS and skeletal muscle atrophy have also attracted considerable research interest. Growing evidence shows that ROS and redox disturbances may represent important signaling events in skeletal muscle atrophy (Powers et al., 2010, 2012). And therefore elucidating the signaling pathways that connect ROS to skeletal muscle atrophy is also an important key to identifying biological targets for therapeutic intervention in skeletal muscle atrophy (Powers, 2014; Mason et al., 2016).

Based on this premise, in this study we aimed to investigate the involvement of ROS in skeletal muscle atrophy from the perspective of gene regulation, and further examine the therapeutic effects of antioxidants on skeletal muscle atrophy. Microarray analysis was used to validate the expression changes of ROS production-regulator genes during denervation-induced skeletal muscle atrophy. Our previous studies showed that pre-treatment with PQQ prevented neurons from injury through inhibiting intracellular ROS production (Qin et al., 2015; Zhang et al., 2016). N-acetylcysteine (NAC) is an ROS scavenger, which could enhance the SOD activity (Sayin et al., 2014). Therefore, we chose pyrroloquinoline quinone (PQQ, a naturally occurring antioxidant) and N-acetyl-L-cysteine (NAC, a clinically used antioxidant) to treat the denervated mouse muscle or fasted C2C12 myotubes (myotubes differentiated from C2C12 myoblastic cells), respectively, for evaluating the effects of antioxidant therapy on skeletal muscle atrophy through inhibition of ROS production.

## Materials and methods

### Animal treatments

Animal experiments were carried out in accordance with the institutional animal care guidelines of Nantong University and approved by the administration Committee of Experimental Animals, Jiangsu Province, China. Male ICR mice with similar initial body weights (about 20 g) were used for all the experiments, and routinely maintained under the same conditions (temperature 22°C, 12:12-h light-dark cycle) with free access to standard laboratory rodent chow and water.

Mice were randomly divided into one control group and three experimental groups (*n* = 12). Animals in the three experimental groups were subjected to unilateral sciatic nerve transection. Briefly, each animal was anesthetized by an intraperitoneal injection of complex narcotics, and the sciatic nerve was exposed through an incision on the lateral aspect of the mid-thigh of the left hind limb. A 1 cm long segment of sciatic nerve was then resected at the site just proximal to the division of tibial and common peroneal nerves, and the incision sites were then closed (Li et al., 2013; He et al., 2016). Then these animals were performed with daily intraperitoneal injection of saline, PQQ (5 mg/kg) in saline, or NAC (100 mg/Kg) in saline, respectively. PQQ and NAC were purchased from Sigma-Aldrich (St. Louis, MO). Animals in the control group received sham-operations and then were injected with the same amount of saline daily. All the treatment lasted 14 days. At the end of the treatment period, the animals were euthanized under anesthesia, and their soleus muscles were dissected, removed, rapidly frozen on liquid nitrogen, and stored at −80°C for subsequent experiments.

### Cell culture and treatments

Briefly, C2C12 cells (a myoblastic cell line) were obtained from the American Type Culture Collection, and were grown in high-glucose Dulbecco's modified Eagle's medium (DMEM) supplemented with 10% fetal bovine serum (FBS), 100 U/ml of penicillin, and 100 μg/ml of streptomycin in a 10% CO_2_ humidified atmosphere at 37°C. To induce differentiation, the cells were incubated in differentiation medium (2% horse serum in DMEM) (Invitrogen, Waltham, MA) for 7 days (Sun et al., 2014b). The C2C12-differentiated myotubes were subjected to fasting (nutritional deprivation, ND) for 12 h in amino acid free and serum free Hank's balanced salt solution. Then the fasted myotubes were incubated for 12 h with NAC (5 mM) or PQQ (20, 40, 80 or 160 μM) dissolved in Hank's balanced salt solution as described previously (Beharry et al., 2014). After treatment, the C2C12 myotubes were examined by morphometric or biochemical assays.

### Microarray analyses and bioinformatics analysis

The mouse soleus muscles, which had been harvested at different times (0, 0.25, 0.5, 3, 6, 12, and 24 h, and 3, 7, 14, 21, and 28 days after sciatic nerve transection), were homogenized for RNA extraction using RNeasy Mini Kit (Qiagen, Valencia, CA) according to the manufacturer's instructions. Microarray analysis was performed using an Agilent SurePrint G3 Rat GE (8 × 60K, Design ID: 028279) on these muscle samples. All assays were performed in at least three biologically independent samples. Microarray analysis was performed on an Agilent Gene Chip platform and scanned by Agilent Scanner G2505C (Agilent Technologies). Data were extracted from scanned images using Agilent Feature Extraction Software (version 10.7.1.1, Agilent Technologies). The raw data were normalized by Genespring Software (version 13.1, Agilent Technologies). The weighted gene co-expression network analysis (WGCNA) was performed, which permits identification of modules of highly co-expressed genes whose grouping reflects shared biological functions and key functional pathways, as well as key hub genes within the modules (Liu et al., 2017).

### Western blot analysis

Western blot analysis was performed as described previously (Sun et al., 2009). The C2C12 myotubes or soleus muscles were homogenized in a radio immunoprecipitation assay buffer (50 mM Tris-HCl pH 7.4, 150 mM NaCl, 5 mM EDTA, 1% Nonidet P-40, 1% sodium deoxycholate, 0.1% SDS/1% aprotinin, 50 mM NaF, and 0.1 mM Na_3_VO_4_) and quantified with Bio-Rad Protein Assay Kit (Bio-Rad, Hercules, CA). Equal amounts of total protein per lane were subjected to SDS-PAGE, and transferred onto polyvinylidene difluoride membranes (Millipore Corp), which were blocked with 5% non-fat dry milk in Tris-buffer saline (TBS) followed by incubation with primary antibodies: mouse anti-MHC (1:3,000) (R&D Systems, Minneapolis, MN), rabbit anti-MAFbx (1:3,000) (Abcam, Cambridge, UK), rabbit anti-MuRF-1 (1:3,000) and mouse anti-tubulin (1:3,000) (Santa Cruz, Santa Cruz, CA) at 4°C overnight. Then, the membrane was probed with the appropriate horseradish peroxidase-coupled secondary antibody. Immunoactive bands were visualized by enhanced chemiluminescence (Thermo Scientific, Park Ellisville, MO). Densitometry analysis was determined by scanning immunoreactive bands, and intensity values were obtained for further normalization against loading control.

### Fiber CSA and myotube diameter measurement and quantification

The fiber CSA of the soleus muscle was detected by using laminin staining. Briefly, fresh-frozen soleus muscles were sectioned on cryostat with 10-μm thickness. The cryosections were placed on glass slides, and incubated overnight with laminin (Abcam) at 4°C. Slides were mounted and imaged by fluorescence microscopy. The fiber CSA was determined through a blinded analysis with the ImageJ software (NIH, Bethesda, MD). Six animals were used to determine the muscle CSA. Five randomly captured muscle images were chosen from each animal. The fiber CSA value is expressed as the mean ± SD.

The myotube diameter was detected by using MHC staining. Briefly, C2C12 cells were grown and differentiated on glass coverslips and then fixed, permeabilized, and incubated for 1 h with mouse anti-MHC (1:3,000) (R&D Systems). After antibody removal and several washes, the slips were incubated for 30 min with 1:100 affinity-purified Alexa Fluor dye-conjugated goat anti-mouse antibody (Life Technologies, Carlsbad, CA). The slips were photographed with the fluorescence microscopy. Myotube diameter was measured at least 100 myotubes using ImageJ software. The myotube diameter was determined at three points along the length of the myotube in a blinded fashion, and the average diameter per myotube was expressed as the mean of three measurements. Myotubes were defined as all multinucleated cells positive for the MHC stain and containing at least three nuclei (Abrigo et al., 2016).

### Determination of ROS

The ROS level in myotubes or in soleus muscles was measured by using dichlorodihydrofluorescein diacetate (DCFH-DA) or dihydroethidium (DHE) staining (Sigma-Aldrich), respectively.

DCFH-DA is one of the most widely used techniques for directly measuring the redox state of a cell (Eruslanov and Kusmartsev, 2010). DCFH-DA transforms into the fluorescent compound dichlorofluorescein (DCF) upon oxidation by ROS. Briefly, myotubes were washed with phosphate-buffered saline (PBS) and fresh DMEM without phenol red, and incubated with 10 μM of DCFH-DA for 15–30 min in the dark at room temperature. The cells were immediately analyzed, and ROS production was measured by an increase in DCF fluorescence. DCF fluorescence was measured at an excitation wavelength of 488 nm and an emission wavelength of 519 nm.

To perform DHE staining on mouse soleus muscles, animals were perfused with 20 ml saline containing DHE (10 μM) about 20 min, and then with 30 ml 4% paraformaldehyde in 10 mM PBS (4°C, PH 7.40). Following, soleus muscles were taken, sectioned, and examined under an Axio Imager microscope (ZEISS, Tokyo, Japan). DHE fluorescence was measured at an excitation wavelength of 535 nm and an emission wavelength of 610 nm. The numbers of DHE-positive nuclei and the total nuclei were counted in five microscopic fields per section. Five muscle sections from each mouse were counted to determine the percentage of DHE-positive nuclei.

### Statistical analysis

All data are expressed as means ± SD. Student's *t*-test and One-way ANOVA was used to compare differences between groups. All statistical analyses were conducted with a SPSS Software Version 17.0 (SPSS Inc., Chicago, IL). *P* < 0.05 was considered as statistically significant.

## Results

### Gene expression changes in skeletal muscle during denervation-induced muscle atrophy

**Figure 1:**
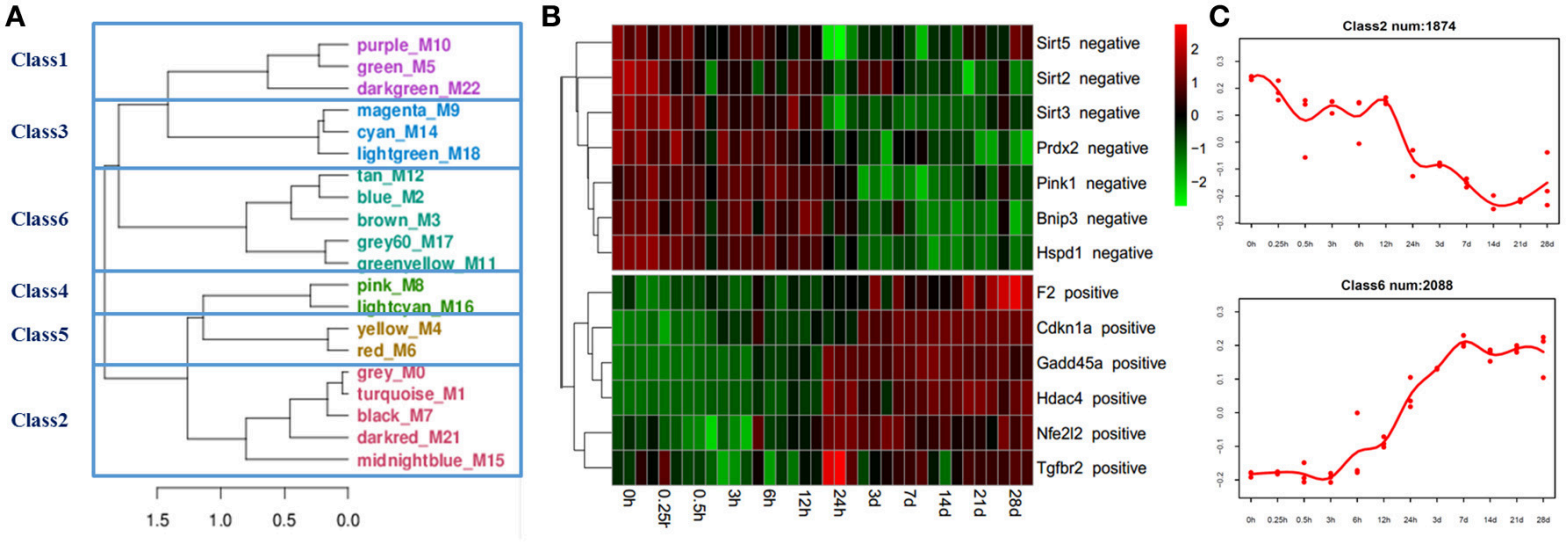
Bioinformatics analysis of gene expression in skeletal muscles identifies distinct modules of co-expression genes. **(A)** The gene modules generated by weighted gene coexpression network analysis (WGCNA) using microarrays data from 36 skeletal muscle samples. The 20 co-expression modules were obtained and clustered into 6 classes by cluster merging module. **(B)** Heatmap of ROS production-related genes, positive regulators for ROS production were gradually up-regulated, and negative regulators for ROS production were gradually down-regulated. **(C)** The negative and positive regulators for ROS production were mainly distributed in classes 2 and 6, respectively.

Microarray analysis was applied to investigate expression changes of ROS production-related genes in the mouse soleus muscle after sciatic nerve transection. A total of 36 microarrays were used to analyze gene expression profiles for muscle samples harvested at 0, 0.25, 0.3, 3, 6, 12, and 24 h, and 3, 7, 14, 21, and 28 days post nerve injury, respectively. One microarray data we further performed the WGCNA analysis, a method that permits identification of modules of highly co-expressed genes whose grouping reflects shared biological functions and key functional pathways as well as key hub genes within the modules (Chandran et al., 2016). Then, consensus network analysis was used to define 20 robust and reproducible co-expression modules during denervation-induced skeletal muscle atrophy. These modules were clustered into 6 classes by the cluster merging module (Figure 1A).

ROS production-related genes were mainly distributed in classes 2 and 6. The gene expression of positive regulators for ROS production, such as prothrombin (F2), cyclin-dependent kinase inhibitor 1A (Cdkn1a), growth arrest and DNA-damage-inducible protein 45 (Gadd45a), histone deacetylase 4 (hdac4), nuclear factor erythroid derived 212 (Nfe212), transforming growth factor beta receptor 2 (Tgfbr2), etc., were up-regulated in the denervated mouse soleus muscle. On the contrary, the gene expression of negative regulators for ROS production, such as sirtuin-2 (Sirt2), sirtuin-3 (Sirt3), sirtuin-5 (Sirt5), peroxiredoxin-2 (Prdx2), PTEN-induced putative kinase 1 (Pink1), etc., were down-regulated in the denervated mouse soleus muscle (Figures 1B,C).

### An increased ROS level in skeletal muscle during muscle atrophy

**Figure 2:**
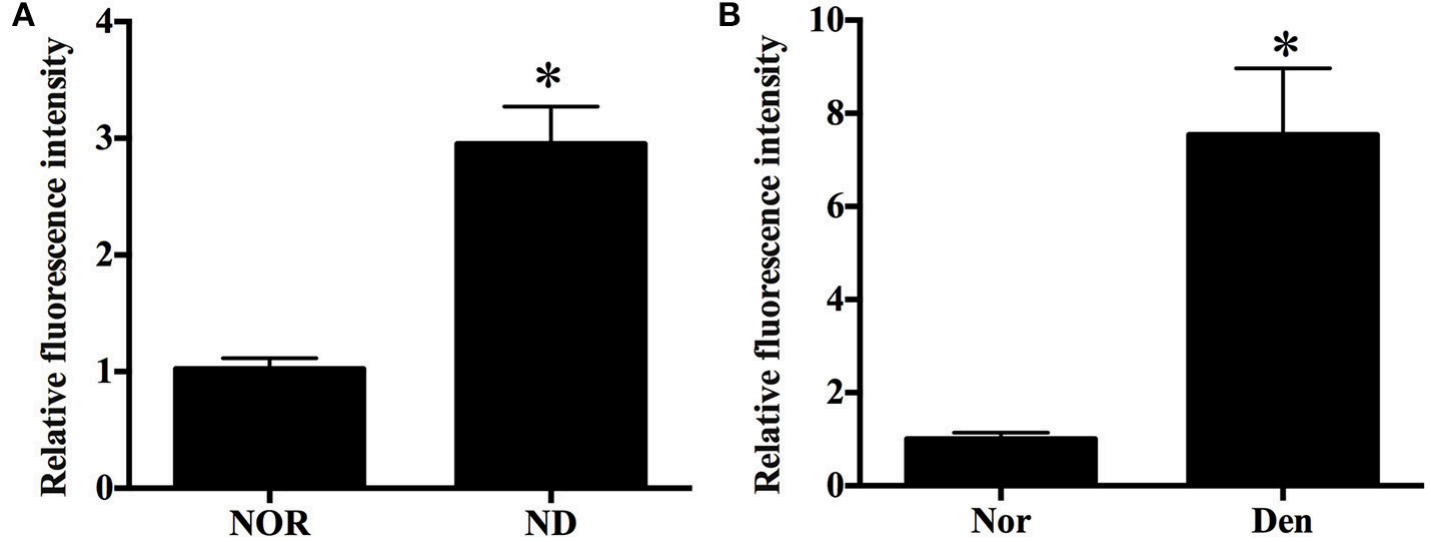
Histograms showing an increased ROS level in **(A)** C2C12 myotubes suffering from fasting (nutrient deprivation, ND) or **(B)** mouse soleus muscles atrophied by denervation (Den) as compared to that in **(A)** C2C12 myotubes or **(B)** mouse soleus muscles without exposure to fasting or denervation stimulation (Nors, serving as respective controls) respectively. ^*^p < 0.05 vs. Nor.

ROS play an important role in inducing several forms of skeletal muscle atrophy. In this study, ROS generation in denervated mouse soleus muscles and fasted C2C12 myotubes was assessed using DCFH staining and DHE staining, respectively. An increased ROS level in two kinds of samples was found during denervation- or fasting-induced atrophy (Figure 2).

### Antioxidant effects on fasting-induced muscle atrophy

**Figure 3:**
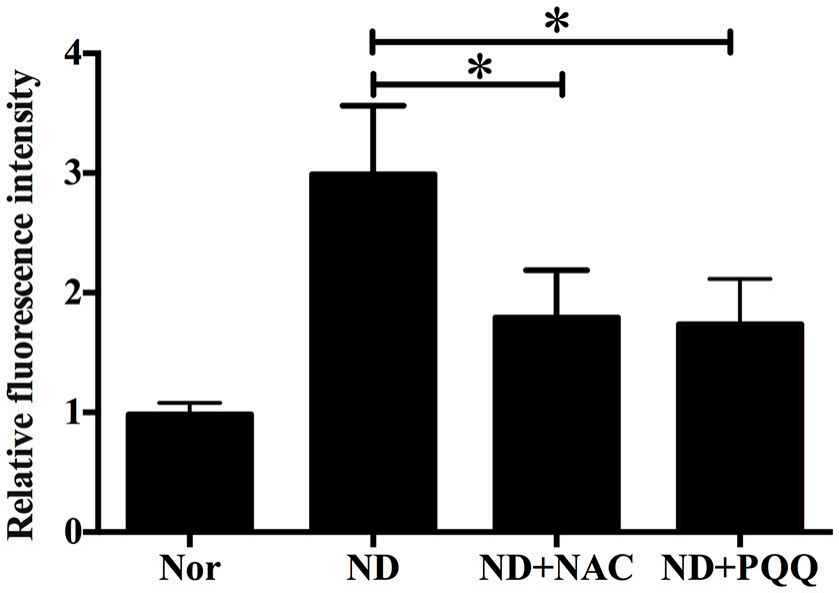
After fasted C2C12 myotubes were incubated with Hank's balanced salt solution for 12 h in the absence or presence of 5 mM NAC or 80 μM PQQ, DCF staining was performed to determine the ROS level in different samples, including normal myotubes (Nor, without exposure to atrophic stimulation and antioxidant treatment), fasted C2C12 myotubes (ND), fasted C2C12 myotubes treated with NAC (ND+NAC), and fasted C2C12 myotubes treated with PQQ (ND+PQQ). Histogram comparing the ROS level (as expressed by DCF fluorescence intensity) among different myotube samples. ^*^p < 0.05.

**Figure 4:**
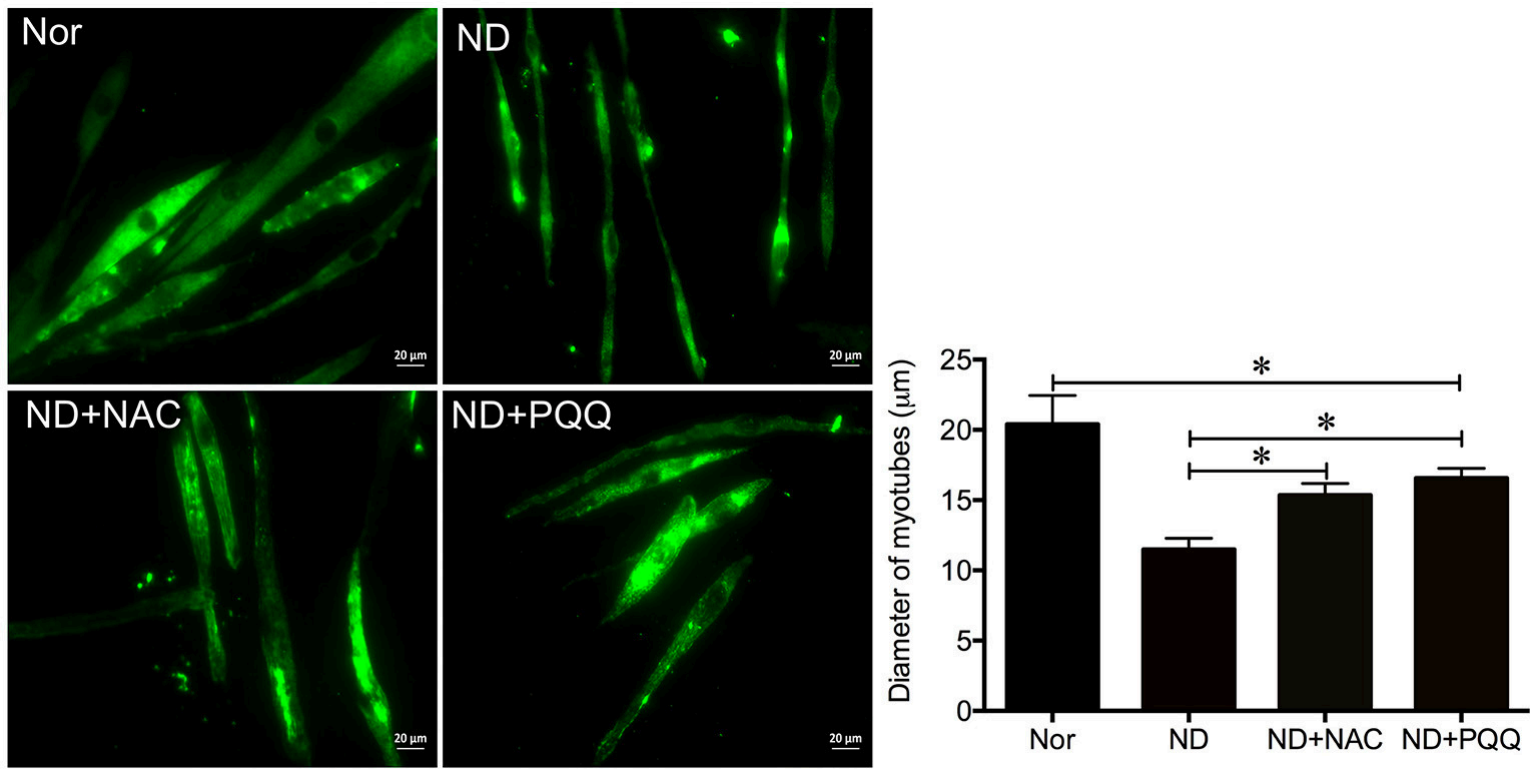
After fasted C2C12 myotubes were incubated with Hank's balanced salt solution for 12 h in the absence or presence of 5 mM NAC or 80 μM PQQ, MHC staining was performed to quantify the diameter of different myotube samples, including normal C2C12 myotubes (Nor, without exposure to atrophic stimulation and antioxidant treatment), fasted C2C12 myotubes (ND), fasted C2C12 myotubes treated with NAC (ND+NAC), and fasted C2C12 myotubes treated with PQQ (ND+PQQ). Histogram **(Right)** comparing the myotube diameter among different myotube samples, where the myotube diameter value is expressed as the mean ± SD from three independent experiments. ^*^p < 0.05. Also shown **(Left)** are representative MHC staining images.

**Figure 5:**
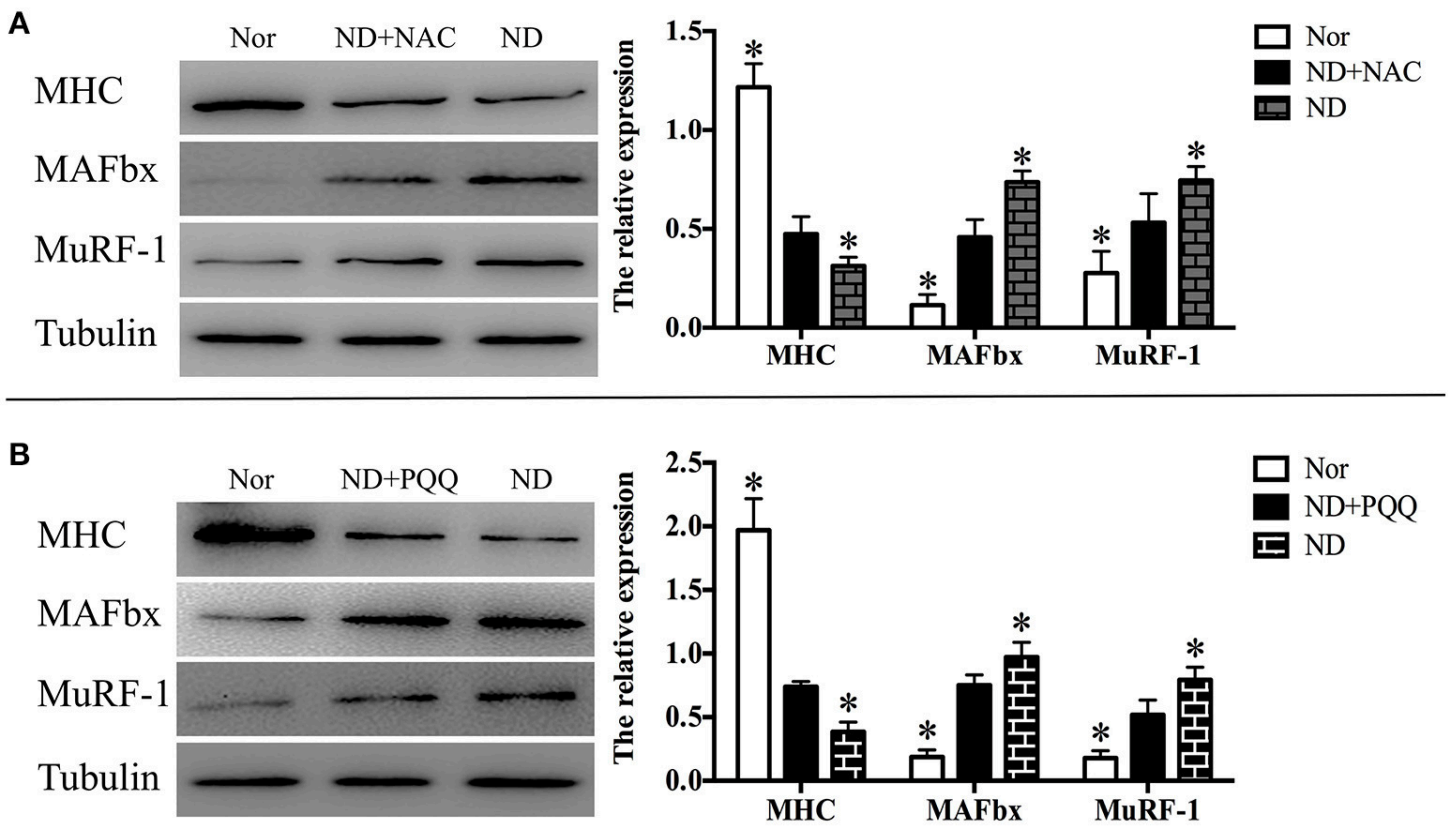
After fasted C2C12 myotubes were incubated with Hank's balanced salt solution for 12 h in the absence or presence of 5 mM NAC **(A)** or 80 μM PQQ **(B)**, Western blot analysis was performed to determine the protein levels of MHC, MuRF-1, and MAFbx in different myotube samples, including normal myotubes (Nor, without exposure to atrophic stimulation and antioxidant treatment), fasted C2C12 myotubes (ND), fasted C2C12 myotubes treated with NAC (ND+NAC), and fasted C2C12 myotubes treated with PQQ (ND+PQQ). Representative Histogram (right) comparing the MHC, MuRF-1, and MAFbx levels among different myotube samples, and ^*^p < 0.05 vs. ND+NAC **(A)** or ND+PQQ **(B)**, respectively. Also shown (left) are representative Western blot images.

The fasted C2C12 myotubes were treated with NAC or PQQ, respectively. For selecting an optimal PQQ concentration, different concentrations (20, 40, 80, or 160 μM) of PQQ were tried, and PQQ at 80, or 160 μM was found to inhibit the generation of ROS without significant difference in the inhibitory effect between the two concentrations (Supplementary Figure S1). Therefore, 80 μM PQQ was used for the ensuing experiments. Treatment with NAC (5 mM) or PQQ (80 μM) significantly reversed the increase in ROS production (Figure 3) and prevented the decrease in myotube diameter (Figure 4). Meanwhile, treatment with NAC or PQQ alleviated the decrease in MHC level, and inhibited the increase in MAFbx and MuRF-1 levels (Figure 5). These results suggested that the increased ROS were associated with fasting-induced muscle atrophy and ROS inhibition could retard muscle atrophy.

### Antioxidant effects on denervation-induced muscle atrophy

**Figure 6:**
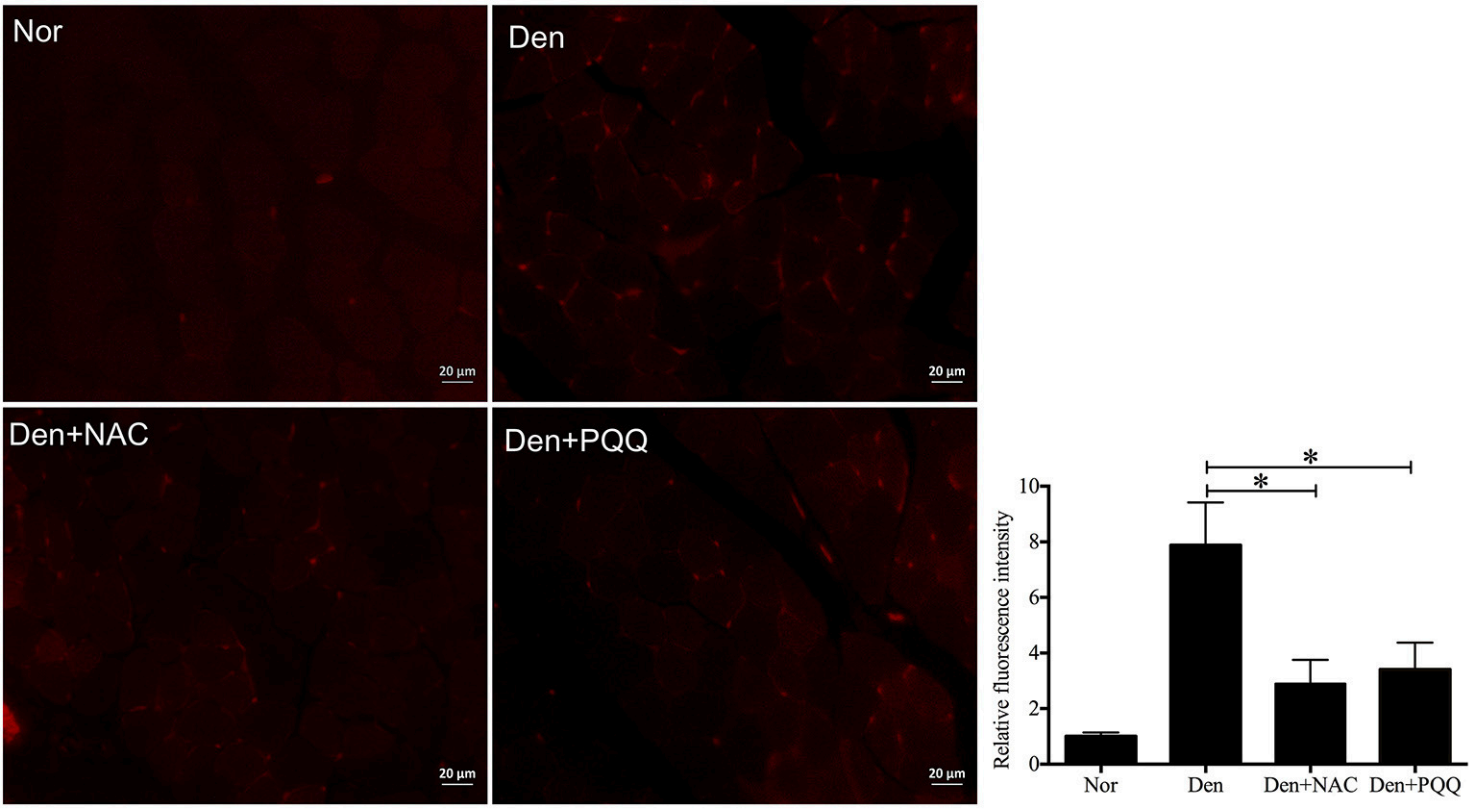
After mice with denervation-induced soleus muscle atrophy had been injected with saline vehicle or saline vehicle plus NAC (100 mg/kg) or PQQ (5 mg/kg) for 14 days, the mouse soleus muscle was harvested for determining the ROS level using DHE staining. Different muscle samples were harvested from mice receiving sham-operation and further saline treatment (Nor, serving as control), mice receiving sciatic nerve transection and further saline treatment (Den), mice receiving sciatic nerve transection and further saline plus NAC treatment (Den+NAC), and mice receiving sciatic nerve transection and further saline plus PQQ treatment (Den+PQQ), respectively. Histogram (right) comparing the ROS level (as expressed by DHE fluorescence intensity) among different muscle samples (*n* = 6). ^*^p < 0.05. Also shown (left) are representative DHE staining images.

**Figure 7:**
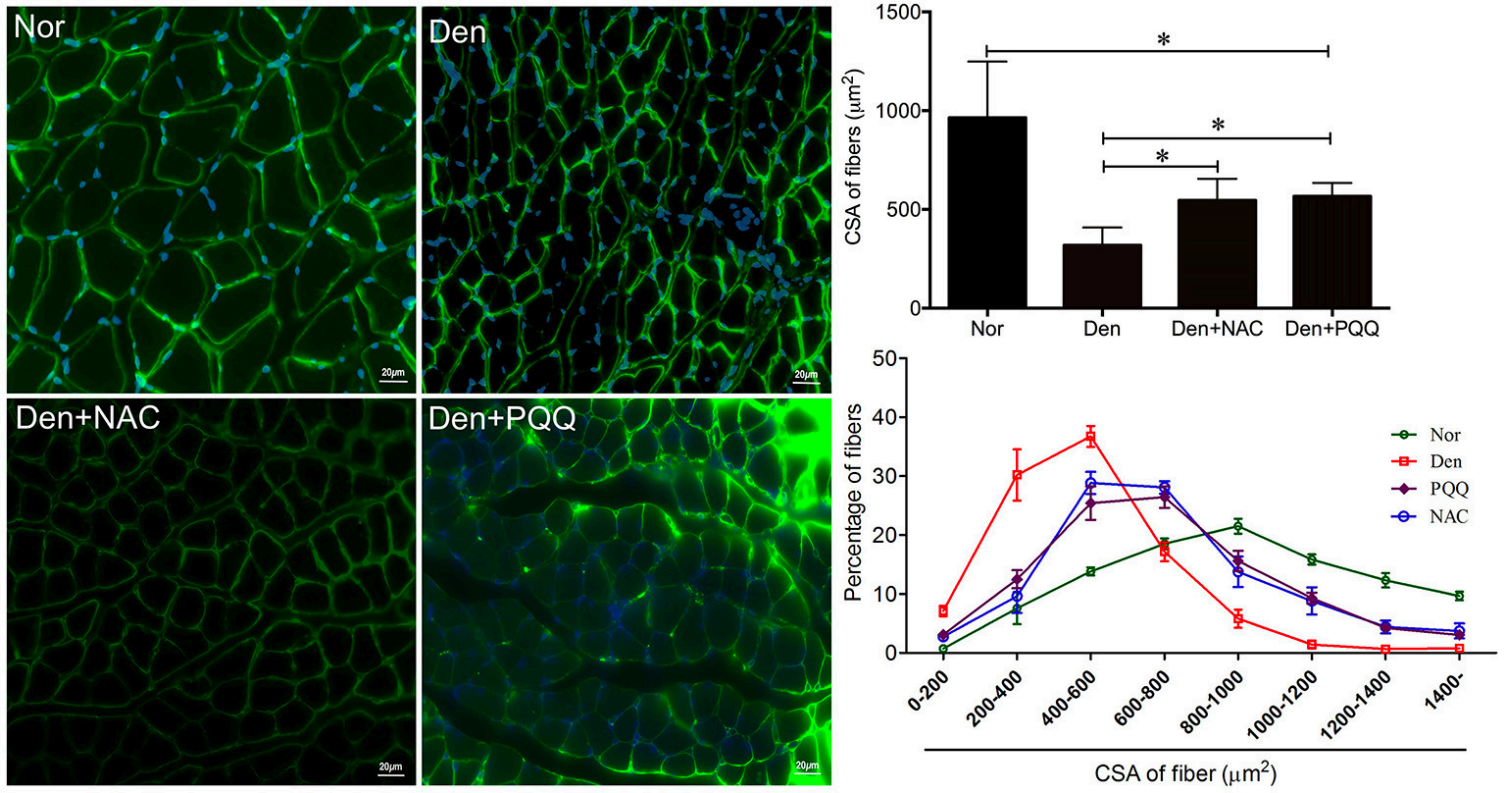
After mice with denervation-induced soleus muscle atrophy had been injected with saline vehicle or saline vehicle plus NAC (100 mg/Kg) or PQQ (5 mg/kg) for 14 days, the mouse soleus muscle was harvested for determining the fiber CSA. Different muscle samples were harvested from mice receiving sham-operation and further saline treatment (Nor, serving as control), mice receiving sciatic nerve transection and further saline treatment (Den), mice receiving sciatic nerve transection and further saline plus NAC treatment (Den+NAC), and mice receiving sciatic nerve transection and further saline plus PQQ treatment (Den+PQQ), respectively. Histogram **(Right)** comparing the fiber CSA (upper: absolute value, and lower: distribution frequency) among different muscle samples, where the fiber CSA value is expressed as the mean ± *SD* from three independent experiments (*n* = *6*). ^*^p < 0.05. Also shown **(Left)** are representative laminin staining images.

**Figure 8:**
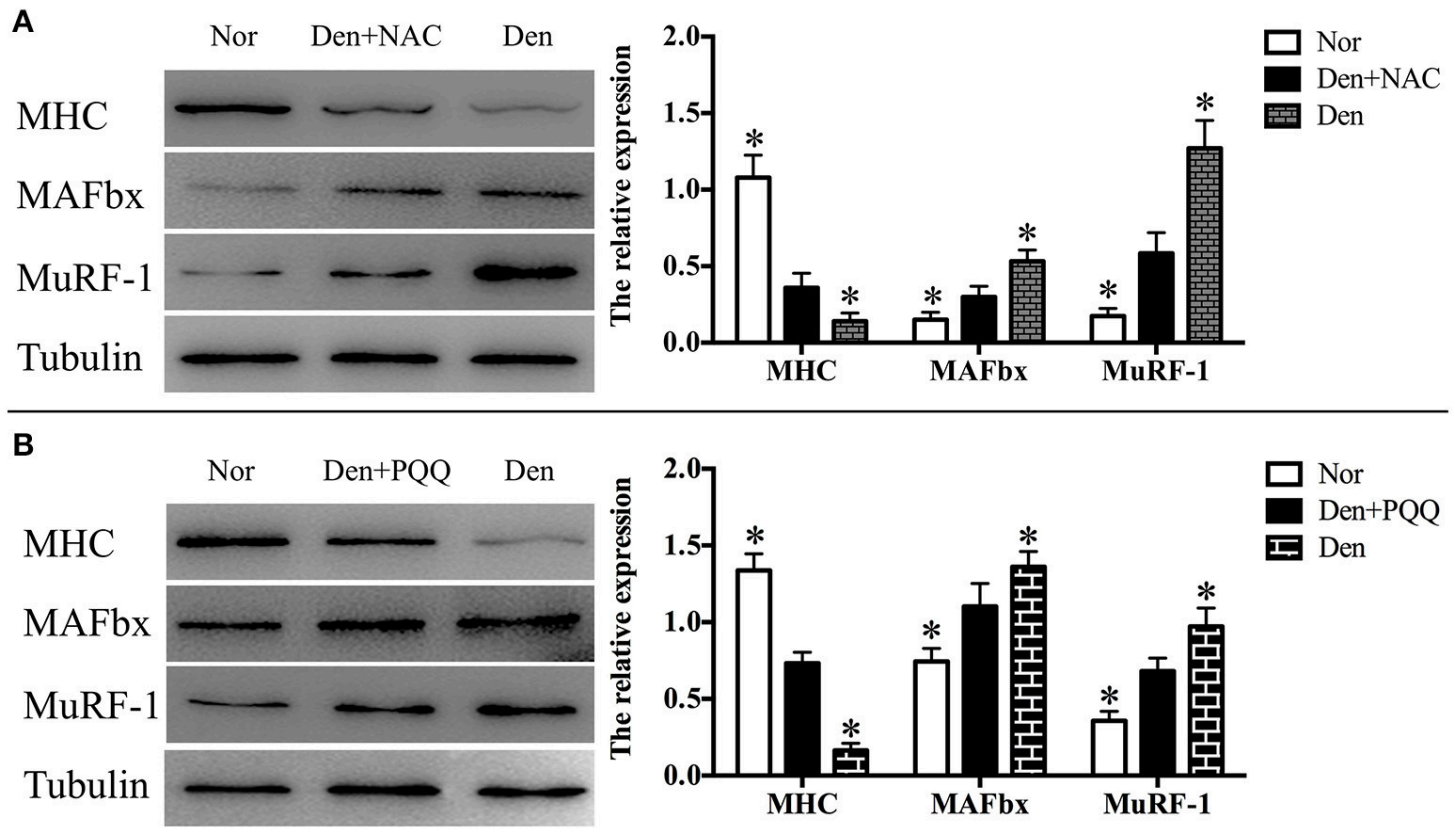
After mice with denervation-induced soleus muscle atrophy had been injected with saline vehicle or saline vehicle plus NAC (100 mg/kg, **A**) or PQQ (5 mg/kg, **B**) for 14 days, the mouse soleus muscle was harvested to undergo Western blot analysis. Different muscle samples were harvested from mice receiving sham-operation and further saline treatment (Nor, serving as control), mice receiving sciatic nerve transection and further saline treatment (Den), mice receiving sciatic nerve transection and further saline plus NAC treatment (Den+NAC), and mice receiving sciatic nerve transection and further saline plus PQQ treatment (Den+PQQ), respectively. Histograms (right) comparing the MHC, MuRF-1, and MAFbx levels among different muscle samples (*n* = 6). ^*^p < 0.05 vs. Den+NAC **(A)** or Den+PQQ **(B)**, respectively. Also shown (left) are representative Western blot images.

The denervated skeletal muscle was treated with NAC and PQQ, respectively. In brief, mice with sciatic nerve injuries were injected daily with saline as a vehicle or with saline as a vehicle in addition to NAC (100 mg/Kg) or PQQ (5 mg/ kg) for 14 days, and then atrophied mouse soleus muscles were harvested to undergo morphological and biochemical measurements. Treatment with NAC or PQQ significantly reversed the increase in ROS production (Figure 6) and prevented the decrease in muscle fiber CSA (Figure 7) as compared to treatment with vehicle alone. Likewise, treatment with NAC or PQQ also significantly alleviated the decrease in MHC level and inhibited the increase in both MAFbx and MuRF-1 levels (Figure 8) as compared to treatment with vehicle alone. These results suggested that the increased ROS production was associated with denervation-induced muscle atrophy and ROS inhibition could retard muscle atrophy.

## Discussion

Skeletal muscle atrophy can be induced by a diverse array of external stimuli under different physio-pathological conditions. The endpoints of these stimuli often share a basic feature: an imbalance of muscle protein synthesis and degradation, which in turn results in reduction in muscle mass and fiber size. There probably exist common molecular mechanisms, which enable different atrophic stimuli to cause skeletal muscle atrophy. In other words, transmission of external stimuli to intracellular effector proteins via signaling pathways should be a highly coordinated and regulated process.

Over the past decades, much research efforts have been devoted to the elucidation of these common mechanisms underlying skeletal muscle atrophy. As has been generally accepted, major signaling pathways controlling skeletal muscle growth include the insulin-like growth factor 1–phosphoinositide-3-kinase–Akt/protein kinase B–mammalian target of rapamycin (IGF1–PI3K–Akt/PKB–mTOR) pathway (a positive regulator) and the myostatin–Smad3 pathway (a negative regulator); on the other hand, major signaling pathways controlling skeletal muscle degradation include the UPS and ALP (Schiaffino et al., 2013; Dutt et al., 2015). Additionally, ROS have been involved in the development of muscular dystrophies including Duchenne muscular dystrophy (DMD). Dysfunction of dystrophin, a key protein for muscles, can cause DMD, associated with damaged muscle fibers and muscular atrophy. ROS can activate cytokines including TNF-α through the activation of the NF-κB pathways, which are correlated to dystrophic myocyte. ROS attack sarcolemma and contractile proteins, leading to more muscle dysfunction (Zuo and Pannell, 2015; Zuo et al., 2015a). ROS-mediated regulation of skeletal muscle atrophy has also been widely documented (Powers et al., 2011, 2016), although a very recent report proposed a challenge to the triggering role of ROS in neurogenic muscle atrophy (Fang et al., 2017; Pigna et al., 2017).

To confirm the implication of ROS in skeletal muscle atrophy, in this study, we performed microarray analysis to profile the gene expression pattern in the denervated skeletal muscle. The WGCNA clustering indicated that 20 robust and reproducible co-expression modules were defined during denervation-induced skeletal muscle atrophy, and all the modules were grouped into 6 classes. ROS production-related genes were mainly distributed in class 2 and class 6. The gene expression of some positive regulators of ROS production was gradually up-regulated, and the gene expression of other negative regulators was gradually down-regulated.

Interestingly, the existing knowledge can be used to discuss those differentially expressed genes, which were identified by our microarray analysis, in the denervated muscle sample.

Sirt2, 3, 5, Pink1, and Prdx2 displayed down-regulation during denervation-induced muscle atrophy. Sirt2, 3 and 5 decreases the cellular levels of ROS or promotes the transcription of manganese superoxide dismutase (MnSOD) (Wang et al., 2007; Lin et al., 2013; Pillai et al., 2016; Yang et al., 2016). Pink1 overexpression could suppress ROS formation, and Prdx2 is an antioxidant protein, whose predominant function is to neutralize ROS (Wang et al., 2011; Chien et al., 2013; Avitabile et al., 2014; Duan et al., 2016). Therefore, down-regulation of above identified genes (cataloged into class 2) during denervation-induced skeletal muscle atrophy might be due to their function as a negative regulator of ROS production.

Cdkn1a, Gadd45a, Hdac4, and Nrf2 displayed up-regulation during denervation-induced muscle atrophy. Cdkn1a and Gadd45a could induce ROS production as result of signaling through GADD45-MAPK14(p38MAPK)-GRB2-TGFBR2-TGFβ (Passos et al., 2010; Borodkina et al., 2014). Hdac4 and Nrf2 could significantly reduce ROS production (Liu et al., 2012; Lisk et al., 2013; Zucker et al., 2014; Chen et al., 2015; Du et al., 2015; Fang et al., 2017). Therefore, the above identified genes (cataloged into class 6) were positive regulators of ROS production, and so they were up-regulated during denervation-induced skeletal muscle atrophy. Of course, this study was restricted by the limited outcomes of our microarray analysis, and thus more numerous regulator genes of ROS production during skeletal muscle atrophy needed to be further examined.

After microarray analysis, DCFH-DA and DHE staining showed that denervation- and fasting (nutrient deprivation)-induced skeletal muscle atrophy did increase ROS production in muscle samples. Skeletal muscle atrophy is closely related to a reduced myofibrillar protein content, leading to a decrease in muscle fiber CSA or in myotube diameter (Huang and Zhu, 2016). Meanwhile, high levels of ROS enhance the protein breakdown through increasing the level of atrophy-related protein, such as MuRF1 and MAFbx (Rodney et al., 2016), and so the protein level of MuRF1 or MAFbx can be regarded as a measure for the degree of skeletal muscle atrophy. In this study, we determined the above morphological and biochemical parameters to evaluate the atrophic degree in two different muscle samples.

Since ROS-related signaling pathways were shown to be involved in skeletal muscle atrophy, antioxidant therapy should be tested for its effectiveness in retarding denervation- or fasting-induced skeletal muscle atrophy. Two antioxidants (NAC and PQQ) were selected for treatments of two atrophied muscle samples. NAC has long been used for clinically treating acetaminophen (paracetamol) overdose. Its other therapeutic potentials, including the alleviation of clinical symptoms of cystic fibrosis, have also been noticed (Rushworth and Megson, 2014; Lasram et al., 2015; Moirangthem and Patel, 2017). Although antioxidant effects of NAC in a range of diseases have been increasingly concerned, a consensus has not yet been reached about its acting mechanisms (Rushworth and Megson, 2014). In contrast to NAC, PQQ has been known as a water-soluble, naturally occurring antioxidant (Raghuvanshi et al., 2016) since it was first discovered as the third redox cofactor in bacteria after nicotinamide and flavin in 1964 (Ge et al., 2015). PQQ mediates redox reactions in the mitochondrial respiratory chain, and plays a critical role in scavenging ROS and attenuating oxidative stress (Nakano et al., 2015). Pretreatment of rats with PQQ obviously reduces the generation of ROS after intracerebral hemorrhage probably based on its antioxidant properties (Lu et al., 2015). PQQ also significantly enhances the activities of SOD, catalase, and glutathione peroxidase (Guan et al., 2015).

In this study, two different models of skeletal muscle atrophy were treated with NAC and PQQ, respectively. After treatments, either antioxidant alone significantly reduced the ROS level in the atrophied muscle samples. According to the determination of the muscle fiber CSA or myotube diameter and MAFbx and MuRF-1 levels, the attenuating effects of two antioxidants on denervation- or fasting-induced skeletal muscle atrophy were validated, respectively.

Recently, a growing body of studies attempt to elucidate the acting mechanisms of antioxidant therapy for skeletal muscle atrophy (Barbieri and Sestili, 2012). Among others, a ROS signaling pathway, which is associated with the dysregulation of peroxisome proliferator activated receptor gamma co-activator-1α (PGC-1α), has been proposed. PGC-1α, as a potent transcriptional co-activator, regulates several metabolic processes, including mitochondrial biogenesis and oxidative phosphorylation (Chan and Arany, 2014). One study by Kuo et al. reports that PQQ resists denervation-induced skeletal muscle atrophy by activating PGC-1α and maintaining mitochondrial electron transport chain complexes (Kuo et al., 2015). Sirt3 has been identified as a downstream target of PGC-1α to suppress cellular ROS production and mitochondrial biogenesis (Kong et al., 2010). Here we conceived that down-regulation of Sirt3 expression in the denervated skeletal muscle, as evidenced by our microassay analysis, might be favorable to ROS production through its interaction with PGC-1α. The details of this assumption, however, need to be further clarified.

In summary, this study used microarray analysis to show that the gene expression of positive and negative regulators for ROS production was respectively up-regulated and down-regulated during denervation-induced skeletal muscle atrophy. We also noted that treatment with either of two antioxidants (NAC and PQQ) significantly retarded the development of skeletal muscle atrophy induced by denervation or fasting. Collectively, our results provided further evidence for the involvement of ROS in skeletal muscle atrophy and suggested the therapeutic potential of antioxidants for skeletal muscle atrophy.

## Author contributions

HS and FD designed the study; JQ, QF, TX, CW, LX, LW, XY, and SY performed the experiments; JQ, QF, TX, CW, and LX collected and assembled data; XY, SY, and QZ performed data analysis; FD and QZ provided scientific expertise; HS wrote the manuscript.

### Conflict of interest statement

The authors declare that the research was conducted in the absence of any commercial or financial relationships that could be construed as a potential conflict of interest.
