# Supplementary material for: Mechanistic Role of Reactive Oxygen Species and Therapeutic Potential of Antioxidants in Denervation- or Fasting-Induced Skeletal Muscle Atrophy
Source: Front Physiol. 2018 Mar 14;9:215. doi: 10.3389/fphys.2018.00215 (PMC5861206; doi:10.3389/fphys.2018.00215)
Supplement: Supplementary file 1 [file Image1.PDF]

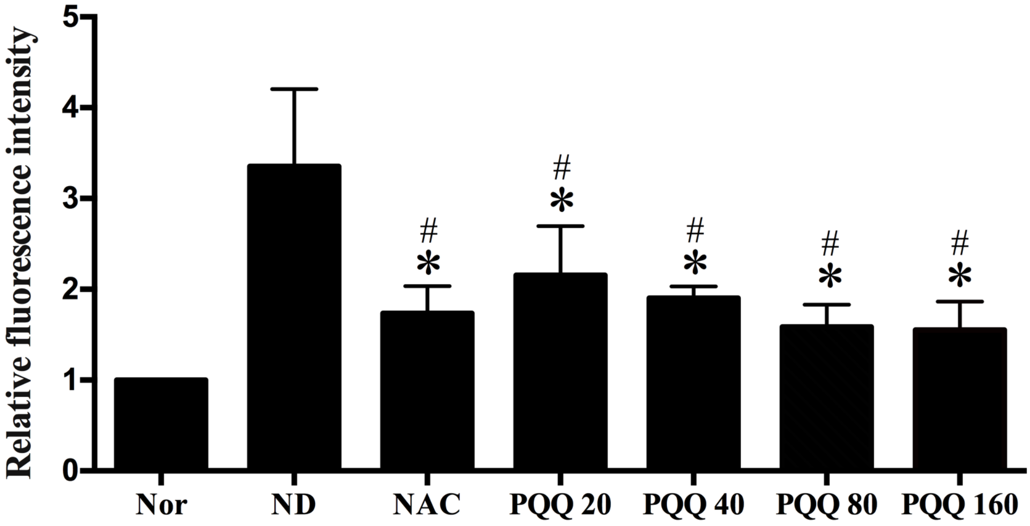

Supplementary Fig S1. After fasted C2C12 myotubes were incubated with Hank's balanced salt solution for 12 h in the absence or presence of 5 mM NAC or different concentrations (20, 40, 80 or 160 μM) of PQQ, DCF staining was performed to determine the ROS level in different samples, including normal myotubes (Nor, without exposure to atrophic stimulation and antioxidant treatment), fasted C2C12 myotubes (ND), fasted C2C12 myotubes treated with NAC (NAC), and fasted C2C12 myotubes treated with PQQ (PQQ 20, PQQ 40, PQQ 80, PQQ 160). Histogram comparing the ROS level (as expressed by DCF fluorescence intensity) among different myotube samples. * p<0.05 versus Nor. $^{\#}$ p<0.05 versus ND (n=6).
